# All nurses need to be research nurses

**DOI:** 10.1017/cts.2017.294

**Published:** 2017-11-16

**Authors:** Patricia Eckardt, Marilyn J. Hammer, Margaret Barton-Burke, Margaret McCabe, Christine T. Kovner, Liza Behrens, Heather Reens, Barry S. Coller

**Affiliations:** 1 Heilbrunn Family Center for Research Nursing, The Rockefeller University, New York, NY, USA; 2 Molloy College, Rockville Centre, NY, USA; 3 Research & Evidence Based Practice, Mount Sinai Hospital, New York, NY, USA; 4 Oncology Nursing Society, Pittsburgh, PA, USA; 5 Nursing Research, Memorial Sloan Kettering Cancer Center, New York, NY, USA; 6 International Association of Clinical Research Nurses, Pittsburgh, PA, USA; 7 Nursing Research, Medicine Patient Services, Boston Children’s Hospital, Boston, MA, USA; 8 Rory Meyers College of Nursing, New York University, New York, NY, USA; 9 School of Medicine, New York University, New York, NY, USA; 10 Program for Person Centered Living Systems of Care College of Nursing, The Pennsylvania State University, University Park, State College, PA, USA; 11 Molloy College, Rockville Centre, NY, USA; 12 The Rockefeller University, New York, NY, USA

**Keywords:** Clinical research nursing, nurse-specific GCP competencies, translational workforce development, NCATS enhance clinical research professionals’ training and qualification (ECRPTQ), clinical research nursing education and professional development program

## Abstract

**Introduction:**

Nurses are critical to the research enterprise. However all nurses are not prepared to participate as members of the research team since education and training in clinical research nursing and nurse-specific Good Clinical Practice are not consistently included in nursing curricula. The lack of nurse education and training in clinical research and Good Clinical Practice leaves research participants vulnerable with a nursing workforce that is not prepared to balance fidelity to protocol and patient quality care and safety.

**Methods:**

A collaborative network of nurses within Clinical and Translational Science Awards and beyond was established to address this education and training need. Over a 2-year period, using expert opinion, Delphi methods, and measures of validity and reliability the team constructed curriculum and knowledge test items.

**Results:**

A pilot modular electronic curriculum, including knowledge pretest and post-tests, in clinical research nursing and nurse-specific Good Clinical Practice competencies was developed.

**Conclusions:**

As the scope and setting of clinical research changes, it is likely that all practicing nurses, regardless of their practice setting or specialty, will care for patients on research protocol, making all nurses, in essence, clinical research nurses. The curriculum developed by this protocol will address that workforce education and training need.

## Introduction

The clinical research nurse (CRN) plays a vital tripartite role in the research enterprise, serving simultaneously as an expert nursing care giver, a member of the scientific team conducting the study, and as the research participants’ advocate who insures that the informed consent process extends throughout the study [1]. In the United States, clinical research nursing as a discipline began under the direction of Ms. Nancy Ellicott at the Rockefeller Institute Hospital in 1910 and then extended out in the 1950s and 1960s through the NIH Clinical Center and the academic medical centers participating in the NIH-supported General Clinical Research Center (GCRC) program [2]. These small specialized units provided focused education to nurses on the requisite skills, including intense observation to identify the earliest signs of adverse events; advanced technical proficiency and fastidious recordkeeping; precise timing of sample collection and sample preparation; and conformity with the complex regulations that have to be met for research data to support applications for new drugs, biologics, and devices from the Food and Drug Administration.

A number of developments highlight the limitations and deficiencies in this previous model that require action to insure that the nursing workforce is prepared to support the evolving changes in the clinical research enterprise. These include the dramatic increase in the number of research participants; the movement of clinical trials out of specialized research units into general hospital care areas and more recently into nonhospital, general medical facilities, and the community in the form of pragmatic trials; the increasingly rigorous standards for initial and ongoing informed consent; the increasing sophistication of the scientific questions under study; the rapid evolution of electronic data recording instruments; and the increasing complexity of achieving compliance with all of the regulations governing the performance of clinical research as codified in the Good Clinical Practice (GCP) standards embodied in the Internal Conference on Harmonization E6.

In recognition of these trends, the NIH discontinued the GCRC program in 2006 and instituted the Clinical and Translational Science Award program, currently housed in the National Center for Advancing Translational Science (NCATS), which radically changed the focus of clinical research from specialized units to the entire academic health center. One feature of this shift that, in our view, has been underappreciated is the need to insure that the broader nursing workforce has the requisite training and skills to function optimally as CRN. Thus, we propose that all nurses need to be research nurses, able to meet the rigorous demands of research protocols, whether they are in a research unit, a general hospital unit, a nursing home, a dialysis center, a community health center, a solo practitioner’s office, or anywhere else where research participants interact within a health care setting or in the broader nonmedical community.

In response to this need, the International Association of Clinical Research Nurses (IACRN) developed the scope and standards for clinical research nursing and these were recognized by the American Nursing Association by their co-publishing the scope and standards with IACRN in 2016 [3]. Building on this milestone, a group of nurse scientists, educators, and leaders have developed a Clinical Research Nursing Education and Professional Development Program, including a comprehensive curriculum in clinical research nursing GCP for nurses and nursing students that consists of 14 modules, each designed to require 30 minutes of study. Ongoing efforts are directed at developing a certification process that uses the scope and standards as the basis of establishing clinical research nursing as an advanced specialty practice. This will insure that there is a cadre of nurse professionals with the specialized training required to fulfill all of the functions of the CRN. These efforts need to be integrated into the excellent Clinical and Translational Science Award workforce development initiatives, most particularly extending the NCATS Enhance Clinical Research Professional Training and Qualification initiative beyond principal investigators and clinical research coordinators to include research nurses [4].

Although the above achievements and ongoing activities are an extremely important first step, they do not go far enough. While nursing school curricula recognize the importance of research and provide valuable training in nursing science, evidence-based practice, application of research findings to practice, and general ethical principles of human research, they lack content on the actual process of clinical and translational research and regulatory requirements [5].

One way to address this deficiency is to introduce CRN education and GCP training into all nursing school curricula. This is a major undertaking that requires expert faculty with knowledge and experience in CRN and GCP, a structured plan integrating CRN and GCP competencies at all levels of nurse education, a sustainable model for skills acquisition of CRN and GCP competencies, and methods for evaluating whether the skills and competencies have been mastered.

To begin this process, the nurse leaders who created the Clinical Research Nursing Education and Professional Development Program modules for CRN also created a single 45 minute module designed to introduce students and entry level nurses to the principles and practice of clinical research nursing in accord with GCP. While much still needs to be done to insure that the entire nursing community has the skills and expertise to serve as CRNs, setting this as a goal is crucial step forward in speeding the development of more new treatments to more patients, and achieving a true learning health care system.
